# Diabetes as a Risk Factor for Breast Cancer

**DOI:** 10.7759/cureus.8010

**Published:** 2020-05-07

**Authors:** Adenike O Eketunde

**Affiliations:** 1 Public Health, University of Massachusetts Lowell, Lowell, USA

**Keywords:** diabetes, type 2 diabetes, breast cancer

## Abstract

Diabetes is one of the most important chronic conditions worldwide, and breast cancer is the most prevalent cancer in women worldwide. Several types of research have been conducted to ascertain the link between diabetes and its potential for increasing the risk of breast cancer. This research aims to determine the relationship between diabetes and breast cancer; patients with diabetes have a higher risk than the general population of developing cancer, and diabetes patients have a higher incidence and mortality of breast cancer. This research also reviewed the relationship between cytokines, the mitogenic effect of insulin-like growth factors, and breast cells. The review includes searching the PubMed database using the keywords “diabetes,” “breast cancer,” “risk factor,” and “premenopausal.” The search returned 53 articles used for review of this article.

## Introduction and background

Diabetes is a medical disease that causes an increase in blood glucose levels. A fasting blood glucose of 126 mg/dL or higher or an oral glucose tolerance test of 200 mg/dL or higher confirms diabetes. The symptoms of diabetes include increase urinating, feeling very thirsty, feeling hungry, and extreme fatigue. Other symptoms include blurry vision, cuts and bruises that are slow to heal, weight loss despite increased food consumption (type 1), and tingling, pain, or numbness in the hands and feet (type 2). All the symptoms of diabetes result from high glucose levels in the blood, affecting different cells, tissues, and organs [1,2].

Cancer is a medical condition that results from the excessive proliferation of unhealthy tissue. This proliferation of cells can be well differentiated (i.e., function as the normal cell) or less similar (i.e., less functional) compared to the cell of origin or no function. The characteristic of cancers is their ability to invade and destroy surrounding tissues and cells; this also includes metastasis. There are different types of cancer affecting a different part of the body or organs varying in severity, prognosis, and mortality. Cancers are abnormal cells that grow out of control ignoring signals to stop dividing or programmed cell death [3].

Breast cancer is caused by malignant cells (cancerous cells) that proliferate excessively in the breast and invade the surrounding tissue or cells and can spread to another area of the body (metastasis). This type of cancer occurs mostly in women and can affect any location in the breast [4]. Cancer of the breast can be caused by other cancer from a different location by a process known as metastasis. Some of the signs of breast cancer include a lump; this cannot completely rule out or rule in breast cancer, even after considering some clinical factors like regularity, irregularity, consistency, firmness, softness, hardness, mobility, immobility, and the like-swelling of either armpit, dimpling of breasts, and changes in the appearance of the nipple. Diagnosis of breast cancer includes a mammogram (also used as a screening test in women older than 40 years), ultrasound, MRI, biopsy, or laboratory tests [3,5].

The research reviews articles from the PubMed database using the keywords “breast cancer,” generating 406,997 hits, then the keywords “breast cancer” and “diabetes,” which resulted in 4,827 hits. The research was further narrowed to “breast cancer,” “diabetes,” and “risk factors” in “premenopausal women” generating 53 articles. 

## Review

Based on the articles used from the database and keyword searches, there is an association between diabetes and breast cancer. Type 2 diabetes is a medical disease that affects more than 7% of adults in developed countries, and up to 10% to 20% of patients with breast cancer have diabetes; the significant risk factors for type 2 diabetes are old age and obesity, which are also risk factors for breast cancer [6]. Studies of 38,000 women reported that 15% of women had diabetes and were more likely to develop an advanced stage of breast cancer compared to women without diabetes [7]. Other studies report that breast cancer risk for patients with diabetes is 20% greater than the risk in patients without diabetes [8,9].

A meta-analysis was undertaken using a random-effects model to investigate the association between diabetes and breast cancer risk and the risk of breast cancer in women with type 2 diabetes was increased by 27%, although the risk was decreased by 16% after adjustment for body mass index (BMI) [9]. 

According to Wolf et al., “three mechanisms have been postulated to associate diabetes with breast cancer: activation of the insulin pathway, activation of the insulin-like growth factor pathway, and regulation of endogenous sex hormones” [6]. Another mechanism postulated is chronic hyperglycemia that could increase the risk of breast cancer known as the Warburg effect [9]. Hyperglycemia is associated with increased level Insulin-like growth factor 1 (IGF-1) and inflammatory cytokines, directly and indirectly influencing cancer cell proliferation, apoptosis, and metastasis [10].

IGF-1 is a mitogenic agent to breast cells, and in the circulation, this mitogen binds with IGF-binding protein 3 (IGFBP-3). Premenopausal women showed increased IGF-1 serum concentrations, decreased IGFBP-3 levels, and increased IGF-1/IGFBP-3 ratios. This IGF-1/IGFBP-3 ratio is essential in determining breast cancer risk coupled with other factors, such as age, hormonal use, and family history [11]. This pathway is significant during neonatal and pubertal growth and works by stimulating cell proliferation and interrupting programmed cell death [12].

Similar to IGF-1, insulin is also a mitogen and plays a significant role in breast cancer, and this might be related to underlying insulin resistance [13]. The expression of the insulin receptor is stimulated by insulin in breast cancer cell lines, and overexpression of it can lead to malignant transformation of breast epithelial cells and activation of oncogenes [14]. Increased insulin resistance can lead to reflex hyperinsulinemia, causing an increase in androgen synthesis and decreased production of estrogen; postmenopausal women are at risk of breast cancer because of estrogen deficiency state [15]. Participants in the Shanghai breast cancer study of women aged 25-64 years between 1996 and 1998 consumed a large amount of soy that was high in isoflavones, which act as weak estrogens and as anti-estrogens. The result suggests that "soy protein intake may negatively modulate the effect of IGF-I and may positively modulate the effect of IGFBP-3 levels on premenopausal breast cancer risk" [14]. Estrogen enhances the effect of IGF-1 levels on breast cancer cell growth and modulates the effect of IGFBP-3 levels on premenopausal breast cancer risk [16].

Overproduction of insulin-like growth factor receptor (IGFR; as seen in patients with diabetes) and mutations in genes encoding these enzymes are in several types of cancer such as colon, breast, and prostate cancers. IGFR overproduction and mutation have become targets for new therapeutic interventions [13]. Type 2 diabetes affects adipocytokines and inflammatory mediators, which lead to an increased risk of breast cancer in premenopausal women [17]. A healthy lifestyle and efficient management of chronic metabolic disease prevent the risk of developing breast cancer and other chronic diseases [17]. The risks associated with breast cancer include the activation of the insulin pathway, activation of the IGF pathway, and regulation of endogenous sex hormones. Type 2 diabetes may be associated with a 10% to 20% excess relative risk of developing breast cancer, and these associations were predominant among women with estrogen receptor-positive breast cancer [18]. There is no relative risk found in women with gestational diabetes [10]. About 50% of breast cancer patients with diabetes are likely to result in death and are at the most significant risk of chemotherapy-related toxicity [19].

Insulin resistance and reduced estrogen production result in a complex metabolic disorder that contributes to the development of cancer in organs with high estrogen demand including breast, endometrium, and ovaries. Insulin resistance leads to a compensatory mechanism that increases the production of androgens and decreases the production of estrogen [15]. Insulin is a potent human hormone regulator and modulates signals at the receptor level [15].

Tumors linked to diabetes may secrete cytokines, especially those that secrete large amounts of interleukin-6, which is associated with insulin resistance and inflammatory signaling throughout the body [20]. In estrogen receptor breast cancer, anti-estrogenic drugs tend to be upregulated by crosstalk between the IGF-1 receptor and the estrogen receptor. In human epidermal growth factor receptor 2 (HER2) receptor-positive breast cancer, there has been increased resistance against drugs (e.g., trastuzumab) used to treat this type of cancer as a result of overexpressing HER2; trastuzumab activity is disrupted by increased expression of IGF-1 receptor [12].

One of the strongest risk factors for breast cancer is high breast cancer density; along with other factors, insulin treatment associated with mammographic density does not imply increased breast cancer risk [21]. Other breast cancer risk factors include nulliparity, obesity, diabetes mellitus, alcohol dependence, smoking, and low levels of physical activity [22].

The relationship between diabetes and breast cancer was confirmed by most studies with large patient populations [23]. The association between diabetes and breast cancer supports the effect of insulin and IGF-1 as a mitogen and also its influences on estrogen hormone levels. However, some studies do not show an increased risk of diabetes in breast cancer patients, and many of these studies had small patient populations [24]. Other studies suggest that breast cancer screening may need to be proactive in women with diabetes to discover cancer at the earliest possible stage [25]. A more aggressive screening plan may include screenings every six to twelve months, along with an annual mammogram and a yearly breast MRI or ultrasound.

Diabetes can lead to end-organ damage and indirectly affects breast cancer by influencing screening and treatment choices, affecting treatment toxicities, and potentially leading to a worse outcome in breast cancer patients. Lifestyle modifications such as weight loss increased physical activity, and dietary changes are attainable in breast cancer populations. Individuals who make lifestyle changes after a breast cancer diagnosis experience several physical and psychological benefits [26]. In breast cancer patients with type 2 diabetes mellitus prior to diagnosis, the mortality rate is increased compared to individuals without diabetes (hazard ratio 1.17). The mortality increased further when type 2 diabetes mellitus was diagnosed at or after the breast cancer diagnosis (hazard ratio 1.39) [27].

## Conclusions

There is a slight increase in the risk of developing breast cancer among women with type 2 diabetes. A decreased level of estrogen as a result of insulin resistance increases the risk of developing cancer in any organ with high levels of estrogen receptors, including breast, endometrium, and ovaries. This review highlights the different mechanisms by which cytokines, IGF-1, and IGFBP-3 levels increase the risk of developing breast cancer. Awareness of these relationships enables healthcare providers to explore ways to support enhanced screening and lifestyle changes to minimize the mitogenic effects of insulin-like growth factors.
